# Do Volatiles Affect Bacteria and Plants in the Same Way? Growth and Biochemical Response of Non-Stressed and Cd-Stressed *Arabidopsis thaliana* and *Rhizobium* E20-8

**DOI:** 10.3390/antiox11112303

**Published:** 2022-11-21

**Authors:** Carina Sá, Diana Matos, Paulo Cardoso, Etelvina Figueira

**Affiliations:** 1 Department of Biology, Campus Universitário de Santiago, University of Aveiro, 3810-193 Aveiro, Portugal; 2 Department of Biology and CESAM, Campus Universitário de Santiago, University of Aveiro, 3810-193 Aveiro, Portugal

**Keywords:** Arabidopsis, *Rhizobium*, volatile organic compounds, biochemical response, growth, metals, 2,3-butanediol, 3-methyl-1-butanol, 2-butanone, cadmium, bacteria, plant, antioxidant response, membrane damage

## Abstract

Plant roots are colonized by rhizobacteria, and these soil microorganisms can not only stimulate plant growth but also increase tolerance to stress through the production of volatile organic compounds. However, little is known about the effect that these plant beneficial volatiles may have on bacteria. In this study, the effects on growth and oxidative status of different concentrations of three volatiles already reported to have a positive influence on plant growth (2-butanone, 3-methyl-1-butanol, and 2,3-butanediol) were determined in *A. thaliana* and *Rhizobium* sp. strain E20-8 via airborne exposure in the presence and absence of Cd. It was expected to ascertain if the plant and the bacterium are influenced in the same way by the volatiles, and if exposure to stress (Cd) shifts the effects of volatiles on plants and bacteria. Results showed the antioxidant activity of the volatiles protecting the plant cell metabolism from Cd toxicity and increasing plant tolerance to Cd. Effects on bacteria were less positive. The two alcohols (3-methyl-1-butanol and 2,3-butanediol) increased Cd toxicity, and the ketone (2-butanone) was able to protect *Rhizobium* from Cd stress, constituting an alternative way to protect soil bacterial communities from stress. The application of 2-butanone thus emerges as an alternative way to increase crop production and crop resilience to stress in a more sustainable way, either directly or through the enhancement of PGPR activity.

## 1. Introduction

In nature, plants coexist with a plethora of microorganisms, establishing interactions that may enhance, inhibit, or have no recognized effect on plants [1]. Plant roots are colonized by rhizobacteria, with some benefiting plants [2]. The beneficial effects include the ability to produce phytohormones, solubilize mineral nutrients, and increase tolerance to stress, with impacts on plant nutritional status, leaf area, chlorophyll levels, soluble leaf protein content [3,4,5], osmotolerance, and production of antioxidant enzymes [6]. These growth-promoting and increasing tolerance effects emphasize the importance of microorganisms in the competition for resources among plants colonizing the same spot, being recognized and used by man throughout the history of agriculture to boost crop productivity.

Some of these interactions occur at a distance, through the production of volatiles [7,8,9,10,11,12]. Volatiles are produced and emitted by plant roots, fungi, bacteria, and protists [10] and can have different biological activities in organisms from different kingdoms [13]. A study performed by Ryu et al. (2003) [7] showed for the first time the importance of volatiles emitted by *Bacillus subtilis* GB03 as agents of growth promotion in *Arabidopsis thaliana*, resulting in the emergence of a new line of research on plant–microorganism interactions. Since then, a series of studies have been conducted to evaluate the impact of volatiles on the growth of *A. thaliana* [14] and other species, such as tobacco [15], wheat [16], or maize [17], to mention just a few. Plant growth-promoting rhizobacteria (PGPR) produce specific volatiles that confer beneficial effects on plant growth through direct stimulation or through induction of systemic resistance [7,18] by regulating phytohormone pathways, photosynthesis, nutrient acquisition, and plant metabolism [19]. Increase in shoot growth, total biomass, seed weight, and early flowering were also reported as effects of PGPR volatiles [5,20].

2,3-Butanediol and acetoin were the first reported volatiles promoting plant growth [7]. More recently, Park et al. (2015) [21] reported 13-tetradecadien-1-ol, 2-butanone, and 2-methyl-n-1-tridecene, produced by *Pseudomonas fluorescens* SS101 and *Bacillus subtilis* SYST2, to enhance plant growth.

However, do bacterial volatiles that promote plant growth also affect soil bacteria?

The expansion of anthropogenic activities led to changes in the environment, such as the increase in soil heavy metal contamination [22,23,24]. Among these, Cd is a very toxic element at low concentrations [25,26] and is classified in seventh place in the 2019 Priority List of Hazardous Substances [27]. Its wide pollution and high mobility in the soil–plant system allows Cd to easily enter the human food chain, posing an increasing threat to the environment and to public health [28,29,30,31]. In the soil solution, concentrations of Cd vary between 0.3 and 8.9 nM [32], but in extremely contaminated sites can exceed 2.7 μM [33]. The primary origin of this contaminant in agricultural soil is the repeated application of phosphate fertilizers [23]. Despite the benefits of these fertilizers, increased concentrations of Cd may affect plants [34], put food safety at risk, and impact the soil microbial communities [35].

Exposure to higher Cd concentrations decreased bacterial abundance in the soil [36]. Moreover, exposure to metals changed the structure and biomass of bacterial communities [37] and impacted their metabolism [38], including the release of volatiles [39,40,41]. In *Rhizobium* cells, Cd decreased growth, caused damage in membranes and proteins, and activated antioxidant and biotransformation mechanisms [42,43,44]. Cd also changed the volatilome of *Rhizobium*, inducing a global increase in the concentration of volatiles both intra- and extracellularly [40]. Plants are also affected by Cd [34,45,46], and although there are no specific transporters in plants for Cd, it can enter the root via the Ca, Fe, Mg, Cu, and Zn channels [47,48]. The utilization of unspecific pathways to enter plants and circulate through the plant vascular system leads to a decrease in essential nutrient uptake and an increase in Cd accumulation, potentiating Cd toxicity [45,46]. Zhou et al. (2017) [24] reported that *Arabidopsis* plants exposed to Cd displayed chlorotic symptoms with biomass loss, chlorophyll reduction, and excessive reactive oxygen species (ROS). Volatiles were described to increase the tolerance of plants to contaminated soils. In a study by Zhou et al. (2017) [24], the release of volatiles by *Bacillus amyloliquefaciens* (strain SAY09) conferred increased Cd tolerance in *A. thaliana* plants. Another *Bacillus amyloliquefaciens* strain (GB03) also showed the ability to release volatiles able to increase plant growth and chlorophyll content and to change the morphological characteristics of *Mentha piperita* plants, increasing their salt tolerance [49]. Under stressful conditions (such as exposure to Cd), are the effects of bacterial volatiles on plants and bacteria shifted?

In order to answer the questions raised, two experiments were set up. Results of these experiments allowed to identify the effect of plant growth-promoting volatiles on plant biochemistry, to ascertain if the effects are the same in bacteria and plants and to confirm if in the presence of stress induced by Cd the effects of these volatiles on *A. thaliana* and *Rhizobium* are maintained or changed.

## 2. Material and Methods

### 2.1. Chemicals

High-purity VOCs (>99%) 2-butanone (78-93-3), 2,3-butanediol (513-85-9), and 3-methyl-1-butanol (123-51-3) were purchased from Sigma-Aldrich (Missouri, St. Louis, MO, USA).

### 2.2. Bacterial Strain

To understand the influence of the volatiles in the presence and absence of CdCl_2_ in soil bacteria, *Rhizobium leguminosarum* strain E20-8 (partial 16S rRNA sequence Genbank accession number KY491644) was used. The tolerance level of this strain to Cd was determined previously [50].

### 2.3. Bacterial Experiment Set-Up

**Figure 1:**
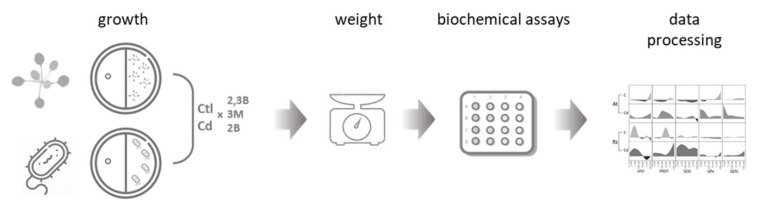
Schematics of the study design. *A. thaliana* and *Rhizobium* were grown in l-plates with VOCs (2,3B-2,3-butanediol, 3M-3-methyl-1-butanol, and 2B-2-butanedione) on one side and plants/bacteria on the other. After incubation (plants—14 days, bacteria—60 h), organisms were weighed (growth) and biochemical parameters (lipid peroxidation—LPO, protein—PROT, superoxide dismutase activity—SOD, glutathione peroxidase—GPx, and glutathione S-transferases—GSTs) were measured, followed by data processing and analysis; artwork own production and from https://biorender.com (accessed on 27 April 2021).

Yeast extract mannitol (YMA) medium [51] supplemented with (100 μM) or without (control) Cd was used to growth the bacterium at different concentrations of the three volatiles (0 nM, 1 nM, 100 nM, 10 μM, 1 mM, and 100 mM). In order to ensure that the influence on bacterial growth was of a volatile nature, center-divided Petri dishes were used as described in previous studies [52,53]. In Section 1, eighteen colonies were inoculated, and in Section 2 a paper disc was set to receive 10 μL of a volatile solution or only the solvent (70% ethanol) as control. For each condition, tests on 3 to 6 plates (replicates) were performed (Figure 1). At the end of the incubation period, the plates were photographed, and colonies were collected. All colonies of a plate were pooled and considered as a sample. After determining the weight of the pooled colonies, they were stored at −80 °C for further analyses.

### 2.4. Plant Experiment Set-Up

*Arabidopsis thaliana* (L.) Heynh. ecotype Columbia (Col-0) seeds grown in our lab (University of Aveiro, Portugal) were used to understand the influence of the three volatiles in the presence and absence of CdCl_2_ in plants. Seeds of *A. thaliana* were sterilized in 70% (*v*/*v*) ethanol, followed by ethanol absolute. Sterilized seeds were transferred to plates containing half-strength Murashige and Skoog medium (MS) medium, pH 5.7, supplemented with 1.5% sucrose and 0.8% agar. Vernalization of seeds was carried out during 2 days at 4 °C. After, using Petri plates with two sections, 3-day-old *A. thaliana* seedlings (5 seedlings/plate) were placed on one section containing MS medium, and in section two, a paper disc receiving 10 μL of a volatile solution at different concentrations (0 nM, 1 nM, 100 nM, 10 μM, 1 mM, and 100 mM) or only the solvent (70% ethanol) was placed on agarized water to hold the disc (Figure 1). Plates were immediately sealed with Parafilm and placed in a growth chamber set for 16 h light/8 h dark, 25 °C and 85% humidity, for 10 days. For each condition, tests on 3 to 6 plates (replicates) were performed. The fresh weight of *A. thaliana* shoots was determined at the end of the assay. The 5 plants grown in the same plate were combined and stored at −80 °C for subsequent analyses.

### 2.5. Biochemical Analyses

#### 2.5.1. Extraction

Plant and bacterial samples were extracted in 50 mM potassium phosphate buffer (pH 7.0): 300 μL for bacterial samples < 0.02 g, 600 μL for bacterial samples ≥ 0.02 g, and 600 μL for plant samples. Bacterial samples were sonicated and centrifuged as described in Matos et al. (2019) [54]. Plant samples were homogenized with a pestle and mortar and centrifuged as described in Lopes et al. (2021) [54]. The supernatant was collected in a new microtube and used immediately or stored at −80 °C for biochemical analysis.

Results of the biochemical parameters (plants and bacteria) were expressed as variation (positive or negative) relative to control (growth without Cd and no volatile influence).

#### 2.5.2. Protein Content

Protein content was determined by the Biuret method (Robinson and Hogden, 1940) as described by Matos et al. (2019) [53]. Biuret reaction solution was added to the sample in a 12:1 (*v*/*v*) ratio After 10 min incubation, samples were read at 540 nm in a microplate reader (Tecan Infinite 200Pro–TECAN, Männedorf, Switzerland). Bovine serum albumin (BSA) was used as a standard.

#### 2.5.3. Oxidative Damage

Lipid peroxidation (LPO) was determined using the method described by Buege and Aust (1978) [55]: 0.5% TBA (thiobarbituric acid) and 10% TCA (trichloroacetic acid) were added to the sample in a 8:1 (*v*/*v*) ratio. After incubation at 96 °C for 25 min, samples were measured spectrophotometrically at 532 nm and nmol of MDA equivalents were calculated (ε = 56 × 10^5^ M^−1^ cm^−1^).

Protein carbonylation (PC) was measured by the 2,4 dinitrophenylhydrazine (DNPH) alkaline method (Mesquita et al., 2014) [56] and modified by Udenigwe et al. (2016) [57] as described in Sá et al. (2020) [52]. To perform the reaction, the sample was mixed with 10 mM DNPH in a 1:1 (*v*/*v*) ratio and incubated for 10 min at room temperature. After, 6 M sodium NaOH was added to the mixture in a 1:4 (*v*/*v*) ratio and incubated for 10 more min. Absorbance was measured at 450 nm and the concentration of the protein carbonyl groups was calculated (ε = 22,308 mM^−1^ cm^−1^).

#### 2.5.4. Antioxidant Enzymes

Superoxide dismutase (SOD) activity was determined by the method described by Beauchamp and Fridovich (1971) [58]. The reaction was performed by mixing the sample with reaction buffer (50 mM Tris-HCl, pH 8.0, 0.1 mM diethylenetriaminepentaacetic acid—DTPA, 0.1 mM hypoxanthine, and 4 μM nitroblue tetrazolium—NBT) and xanthine oxidase (51.6 mU/mL) in a 2:9:1 ratio. After incubation (20 min) with agitation (50 rpm), the absorbance was read at 560 nm and SOD activity determined, taking into account the percentage of NBT reduction compared to control (no sample included in the mixture).

Glutathione peroxidase (GPx) activity was measured by the method described by Paglia and Valentine (1967) [59]. The reaction mixture included the sample, the dilution buffer (50 mM Tris-HCl, pH 7.6, and 5 mM EDTA), 5 mM reduced glutathione, 2 mM cumene hydroperoxide, glutathione reductase (2.5 U/mL), and 2 mM NADPH in a 4:15:8:6:4:3 ratio. The reaction was followed during 20 min, with 15 s intervals, at 340 nm. GPx activity was calculated using the extinction coefficient ε = 0.00622 μM^−1^ cm^−1^.

Glutathione-S-transferase (GST) activity was determined by the method described by Habig et al. (1974) [60]. The reaction buffer (100 mM potassium phosphate buffer, pH 6.5, 10 mM reduced glutathione, and 60 mM 1-chloro-2,4-dinitrobenzene—CDNB) was added to the sample in a 2:1 (*v*/*v*) ratio. The reaction was followed during 20 min, with 15 s intervals, at 340 nm. The activity of GST was determined using the extinction coefficient ε = 9.6 mM^−1^ cm^−1^.

### 2.6. Statistical Analyses

The PRIMER v6 & PERMANOVA+ program (version 6) was used to statistically evaluate the data obtained [61]. The values were subjected to a Monte Carlo test with 9999 permutations. The pseudo-F values in the main tests were evaluated in terms of the significance and when significant (*p* ≤ 0.05) pairwise comparisons were performed between conditions. Significant differences (*p* < 0.05) for the plant and the bacterium between conditions were considered and are identified in supplementary tables, with different lowercase (no Cd) letters to test the hypothesis that when exposed (plant and bacteria) to the same volatile no difference exists among concentrations, uppercase (Cd) letters to test the hypothesis that when exposed (plant and bacteria) to the same volatile in the presence of Cd no difference exists among volatile concentrations, and asterisks to test the hypothesis that under the same concentration of a volatile no difference exists between Cd-exposed and non-exposed organisms (plant or bacteria).

To understand if the biochemical influence that each volatile has on bacteria and plants are related, a principal coordinates ordination (PCO) was performed using the biochemical parameters data. The data were transformed (fourth root), normalized, and used to build a Euclidean matrix. Pearson correlation vectors of biochemical parameters (correlation ≥ 0.7) were included on the PCO graph, allowing the identification of the descriptors that most contributed to the differences observed among the conditions tested. The generated figure can be used to understand which conditions are close to each other and which are different.

## 3. Results

**Figure 2:**
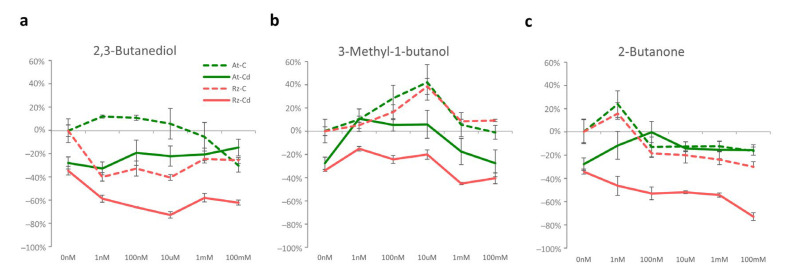
Growth variation relative to control (no Cd and no VOC). *A. thaliana* and *Rhizobium* were simultaneously exposed to 2 Cd conditions (0 and 100 μM) and 6 concentrations (0 nM, 1 nM, 100 nM, 10 μM, 1 mM, and 100 mM) of 2,3-butanediol (**a**); 3-methyl-1-butanol (**b**); and 2-butanedione (**c**) conditions. *A. thaliana* was exposed to VOC and not to Cd (green dashed line); VOC and Cd (green full line). *Rhizobium* was exposed to VOC and not to Cd (pink dashed line); VOC and Cd (pink full line). Values are means of 3–6 replicates + standard errors. For statistical significance, see Supplementary Table S1.

With the aim to determine if plant growth-promoting volatiles (two alcohols and one ketone) induce the same effect on plants (*A. thaliana*) and bacteria (*Rhizobium*), and if this influence is maintained under stress conditions, here simulated by Cd, an experimental design was set up (Figure 2). Both organisms were exposed to a three orders of magnitude concentration of volatiles (nM-mM) in the absence and presence of stress (100 μM Cd). For each condition, growth and biochemical markers were evaluated.

### 3.1. Cd and Volatiles Influence on Growth

The concentration of Cd significantly reduced growth, around 28% for *A. thaliana* and 35% for *Rhizobium* (Figure 2a–c and Supplementary Table S1).

Growth of both organisms was differently influenced by volatiles. In the absence of Cd (dashed lines), two volatiles (3-methyl-1-butanol and 2-butanone) induced similar effects on the plant and the bacterium, with increased growth at low concentrations and with no effect (3-methyl-1-butanol) or with a non-significant negative effect (2-butanone) at higher concentrations (Figure 2b,c). On the contrary, 2,3-butanediol caused opposite effects in the two organisms, increasing *A. thaliana* and decreasing *Rhizobium* growth (Figure 1b). In the presence of Cd (full lines), 3-methyl-1-butanol had an identical effect on both *A. thaliana* and *Rhizobium*, with the three lower concentrations attenuating the negative impact of Cd and the two higher having a small influence on growth (Figure 2b). 2,3-Butanediol and 2-butanone alleviated the negative effect caused by Cd in *A. thaliana* (Figure 2a,c), but both compounds had a mild effect or decreased *Rhizobium* growth compared to sole exposure to Cd.

### 3.2. Cd and Volatile Influence on Plant and Bacterium Biochemistry

#### 3.2.1. Cadmium

**Figure 3:**
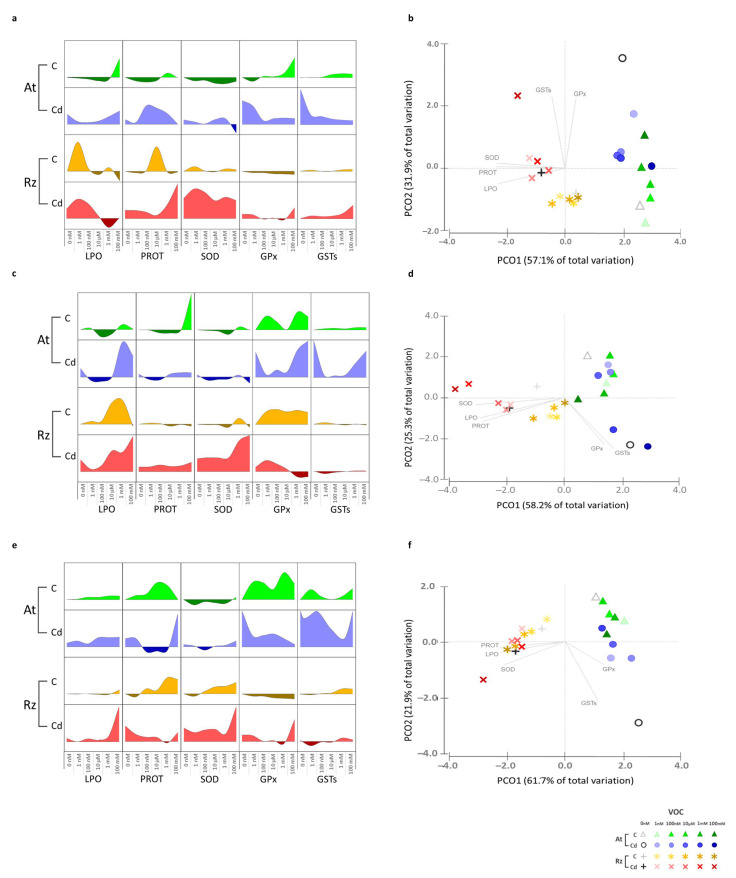
Antioxidant and biotransformation activity and damage (no Cd and no VOC). *A. thaliana* and *Rhizobium* were simultaneously exposed to 2 Cd conditions (0 and 100 μM) and 6 concentrations (0 nM, 1 nM, 100 nM, 10 μM, 1 mM, and 100 mM) of 2,3-butanediol (**a**,**b**); 3-methyl-1-butanol (**c**,**d**); 2-butanedione (**e**,**f**) conditions. VOC on one side of l-plates and plants/bacteria on the other. Variation relatively to control (**a**,**c**,**e**) of lipid peroxidation (LPO), protein (PROT), superoxide dismutase activity (SOD), glutathione peroxidase (GPx), and glutathione S-transferases (GSTs). *A. thaliana* at 0 μM Cd (light green for positive, dark green for negative variation), *A. thaliana* at 100 μm Cd (light blue for positive, dark blue for negative variation), *Rhizobium* at 0 μM Cd (yellow for positive, ocher for negative variation), and *Rhizobium* at 100 μm Cd (pink for positive, red for negative variation). Data range of the same parameter is set to the same scale. Values are means of 4 replicates. For statistical significance, see Supplementary Table S2. Principal coordinates with centroids ordination (PCO) of the biochemical determinants for each condition (**b**,**d**,**f**); detailed color scheme in the figure. Pearson correlation vectors (LPO, PROT, SOD, GPx, and GSTs) (r ≥ 0.70).

The two organisms responded differently to Cd exposure, with *A. thaliana* increasing the activity of GST and GPx and *Rhizobium* increasing SOD, proteins, and LPO (Figure 3).

#### 3.2.2. 2,3-Butanediol (Alone and in Combination with Cd)

Exposure of *A. thaliana* plants to lower concentrations of 2,3-butanediol in the absence of Cd (Figure 3a,b) decreased LPO and the activity of antioxidant enzymes (SOD and GPx). At higher concentrations, protein and LPO levels increased and the activity of GPx and GST also increased, positioning them in increasingly positive values in axis 2 as the volatile concentration increased, bringing them closer to sole exposure to Cd and further away from control. In the presence of Cd, higher concentrations of the volatile reduced GPx, GST (at concentrations ≥ 100 nM), and SOD (mM range) activity. Protein levels were reduced (100 nM to 1 mM), positioning these conditions in the PCO closer to the axis 2 origin, placing them away from sole exposure to Cd and bringing them closer to the control.

In *Rhizobium* (Figure 3a,b), at lower concentrations (1 nM) LPO levels and protein levels increased at intermediate concentrations. However, the overall biochemical response to 2,3-butanediol was not much different from the control, with conditions positioned in the PCO near the origin of axis 1 and on the negative side of axis 2, close to control. The biochemical response of *Rhizobium* to Cd did not significantly change in the presence of 2,3-butanediol. In PCO, most conditions are positioned close to sole exposure to Cd, on the negative side of axis 1 and near the origin of axis 2. Distinct from other conditions, the combined exposure to Cd and 100 mM 2,3-butanediol is found on the positive side of axis 2, evidencing the increased activity of GPx and GST induced by this condition.

#### 3.2.3. 3-Methyl-1-Butanol (Alone and in Combination with Cd)

Low concentrations (<mM) of 3-methyl-1-butanol (Figure 3c,d) reduced LPO, protein levels, and SOD activity in *A. thaliana*, while concentrations in the mM range had the opposite effect, inducing an antioxidant response, positioning them near the axis 2 origin. In the presence of Cd, lower concentrations of 3-methyl-1-butanol (1 nM–10 μM) reduced LPO and protein levels. Enzymatic activity also decreased, especially GPx and GST, placing these conditions further away from sole exposure to Cd and bringing them closer to control. At higher concentrations (mM range), enzyme activity increased to values similar to sole exposure to Cd, and LPO levels were even higher, leading to a biochemical response that positioned these two conditions close to sole exposure to Cd.

In *Rhizobium* (Figure 3c,d), 3-methyl-1-butanol increased LPO levels and GPx activity, placing these conditions on the negative side of axis 2. In the presence of Cd, low concentrations did not influence Cd effects on cell biochemistry. Higher concentrations increased LPO levels (≥10 μM) and SOD activity (≥1 mM) and decreased GPx activity (≥1 mM). The lower concentrations were placed close to sole exposure to Cd and the two higher concentrations further apart at the more negative side of axis 1.

#### 3.2.4. 2-Butanone (Alone and in Combination with Cd)

*A. thaliana* exposed to 2-butanone (Figure 3e,f) slightly increased LPO, augmented proteins, increased the activity of GPx and GST, and decreased the activity of SOD, which induced an overall biochemical response that separated some of these conditions from control in the PCO and positioned them closer to the axis 2 origin. In the presence of Cd, 2-butanone concentrations (100 nM–1 mM) reduced proteins and the activity of GPx and GST. In the PCO, these conditions were positioned further away from sole exposure to Cd and closer to control. However, the highest concentration (100 mM) was closer to sole exposure to Cd (Figure 3f) due to increased protein content and GST activity.

Lower concentrations of 2-butanone had little influence on the biochemistry of *Rhizobium* (Figure 3e,f) in the absence of Cd. However, higher concentrations (range) increased protein levels and SOD and GST activity. In the presence of Cd, lower concentrations (1nM-1 mM) reduced LPO, protein levels, and GPx and GST activity to values similar to the same range of 2-butanone concentrations without Cd. In the PCO, these conditions (concentrations ≤ 1 mM exposed or not to Cd) had a close distribution, on the negative side of axis 1 and near the origin of axis 2. Combined exposure to Cd and the higher concentration of 2-butanone (100 mM) increased LPO levels and SOD and GPx activity, bringing this condition in a more negative position in axis 1 and axis 2 than sole exposure to Cd.

## 4. Discussion

The need to increase agricultural productivity in a more sustainable way has driven research into the promotion of plant growth by rhizobacteria. In the last two decades, the influence of bacterial volatiles on plants has been investigated and several compounds were described as having a positive influence on plant growth. Ryu et al. (2003) [7], based on several experimental results, indicated that 2,3-butanediol is responsible for airborne chemical signaling, triggering growth promotion in *A. thaliana*. Other studies also confirmed that 2,3-butanediol mediate plant-beneficial effects, such as growth promotion and induced systemic resistance (IRS) in tobacco plants [62]. 2,3-Butanediol from the leachates of pine needles activated the resistance of Panax notoginseng to leaf disease infection through ISR and camalexin biosynthesis [63]. In tomato plants, 2,3-butanediol enhanced the transcription of jasmonic acid and salicylic acid responsive genes, increasing tolerance to drought and chilling stress [64]. In our study, 2,3-butanediol also promoted *A. thaliana* growth and had an antioxidant effect at lower concentrations, protecting membranes from damage (LPO decrease) and decreasing antioxidant enzyme activity (SOD and GPx) and cell metabolism (lower soluble protein levels). In the presence of stress (Cd), its antioxidant properties were even more evident, alleviating the toxicity imposed by Cd (lower membrane damage at lower concentrations) and increasing growth, enabling to infer that 2,3-butanediol is beneficial for plants whether they are challenged or not with stress.

Lee et al. (2016, 2019) [65,66] observed that at nM concentrations 3-methyl-1-butanol significantly increased *A. thaliana* fresh weight and chlorophyll content. Nonetheless, Gamboa-Becerra et al. (2022) [67] tested 3-methyl-1-butanol at concentrations in the μM range (25, 50, and 100 μM), and the most positive influence on *A. thaliana* growth was observed at 50 μM. Kong et al. (2021) [68] showed that when *A. thaliana* and Medicago sativa were in stress (iron-limited conditions), the presence of 3-methyl-1-butanol (100 μM) allowed a better plant development and promoted growth; authors related these effects with the mediation of 3-methyl-1-butanol on iron uptake by plants. Results obtained in our study also evidenced the positive effect of 3-methyl-1-butanol on *A. thaliana* growth in the nM to μM range both in the presence and absence of Cd. This volatile decreased antioxidant enzyme activity and membrane damage, evidencing its antioxidant properties, important to protect plants from conditions causing oxidative stress.

2-Butanone was reported to induce Nicotiana tabacum growth [21] and to increase fruit yield in Cucumis sativus [69]. Sidorova et al. (2021) [13] exposed *A. thaliana* to different concentrations of 2-butanone, and at low concentrations (50–100 μM) did not perceive changes in growth, but at higher concentrations (200–400 μM) higher growth was observed. The results of our study evidence an opposite trend, with the lower concentration (1 nM) promoting and the higher concentrations (100 nM to 100 mM) not changing *A. thaliana* growth. The effect of this volatile was especially evident in the presence of Cd, reducing the activity of antioxidant enzymes and membrane damage at concentrations between 100 nM and 1 mM.

The effect of these compounds on bacteria is less known, and their application as a sustainable way to promote crop growth can have an unforeseen impact on the soil microflora. The results obtained in this study allowed us to answer the question whether the bacterial volatiles that promote plant growth also affect bacteria. Results showed that the three compounds tested also influenced bacteria, but the effects varied between compounds, with 3-methyl-1-butanol and 2-butanone promoting *Rhizobium* growth, but 2,3-butanediol decreasing it. On the other hand, even when the growth effects were identical between plants and bacteria, the impact on the biochemical status differed between the plant and the bacterium. In *A. thaliana*, GST and GPx activity was induced as a way to fight the oxidative stress caused by exposure to higher concentrations of the three volatiles. *Rhizobium* showed higher membrane damage (LPO increase), higher changes in metabolism (protein increase), and higher SOD activity to fight the oxidative stress generated by exposure to high volatile concentrations.

Previous studies have demonstrated that not only the presence of volatile compounds can influence the response of organisms in the presence or absence of stress, but the antioxidant response can also vary depending on the volatile and the selected concentration [43,44,53,53]. Sá et al. (2020) [52] reported that limonene at concentrations in the mM range was beneficial when bacterial cells were facing Cd stress but not in non-stressed cells. Matos et al. (2019) [55] showed that aldehydes are more toxic to bacterial cells than alcohols in the presence of stress. These authors also observed that effects were dependent on exposure concentration. Exposure to mM concentrations of alcohols inhibited bacterial growth, and lower concentrations (nM and μM) increased it [53]. Therefore, it is not indifferent to apply any volatile at any concentration that promotes plant growth in an agricultural context. The possible application of volatiles in agricultural fields as a way to promote crop growth should take into account the effects on non-target organisms, such as microbial communities, and if there is knowledge of these effects, the application could be beneficial in several dimensions, either directly to the plant, or indirectly by benefiting PGPR.

This study also showed that the beneficial effects of volatiles were maintained under stress, reducing the negative impact of Cd on *A. thaliana* and answering the second question raised. Under stressful conditions (such as exposure to Cd), are the effects of these volatiles changed in plants and in bacteria? *A. thaliana* triggered mechanisms (GST and GPx activity) to fight the oxidative stress generated by Cd at all concentrations of 2,3-butanediol and 2-butanone and at intermediate concentrations of 3-methyl-1-butanol. Light intensity, radiation, drought, salinity, heat, nutrient deficiency, heavy metals, biocides, nanoparticles, and non-polar organic compounds all induce oxidative stress in plant cells [70,71], and volatiles such as isoprenoids were described to mitigate oxidative stress in plants [72] through the decrease in ROS, lower membrane damage, and lower impact on photosynthesis [73,74]. Vaishnav et al. (2016) [75] showed that bacteria reduced the oxidative damage in plants exposed to NaCl. This effect was linked to the change in the bacterial volatilome induced by salinity, which changed the expression of plant genes coding antioxidant enzymes, and thus reduced lipid peroxidation and phenol oxidation [75,76]. Cappellari et al. (2020) [49] exposed Mentha piperita to NaCl and observed that bacterial volatiles decreased membrane damage.

Our study shows that the effects of the three volatiles on the same bacterium varied. 2-Butanone (a ketone) increased *Rhizobium* tolerance to Cd, but 3-methyl-1-butanol and 2,3-butanediol (both alcohols) were not able to mitigate the oxidative stress induced by Cd in *Rhizobium*. Matos et al. (2019) [55] also reported the divergent effect of different compounds (alcohols and aldehydes) on *Rhizobium* growth, with aldehydes having a higher impact on the cytosol and on membranes. Moreover, a study performed by Cardoso et al. (2017) [40] showed that 2,3-butanediol and 3-methyl-1-butanol increased in *Rhizobium* cells exposed to higher Cd concentrations, while 2-butanone decreased, which could be due to 2-butanone being a precursor of 2,3-butanediol. Our results evidence that at lower concentrations, 2,3-butanediol was not able to induce the antioxidant enzymatic response, with impacts on membrane damage and growth that were more detrimental than Cd alone, but at the highest concentration the increase in GPx and GST activity was able to reduce damage but not to increase growth, which remained lower than in the sole exposure to Cd. Our results also show that 3-methyl-1-butanol had little influence on Cd toxicity at lower concentrations, but at higher concentrations the additivity of the volatile and Cd toxicities increased cellular damage, which was not prevented by the increase in SOD activity. 2-Butanone alleviated Cd stress, with the biochemical status of bacterial cells at lower concentrations being identical to cells not exposed to Cd; however, at the highest concentration GPx activity decreased, and although SOD activity increased it failed to prevent cell damage and growth was lower than in cells only exposed to Cd.

The importance of the antioxidant enzymatic response in the reduction in oxidative stress and protection of organisms from oxidative damage is well known. The three volatiles studied here evidenced antioxidant activity in plants exposed to Cd, reducing its impact on cellular metabolism and increasing tolerance to this toxic metal. Therefore, the volatiles studied here can have an important role in the induction of tolerance mechanisms in plants to pollutants generating oxidative stress. Effects on bacteria were less positive, with 2,3-butanediol and 3-methyl-1-butanol increasing Cd toxicity, but 2-butanone was able to protect bacteria from Cd stress, opening good perspectives to use this volatile in field applications, as it appears to have positive effects on both plants and soil bacteria.

## 5. Conclusions

This study shows that volatile bioactivity differed between plants and bacteria and that this bioactivity is influenced by environmental conditions (e.g., Cd stress), highlighting the potential for application of volatiles in different contexts. In agricultural systems, volatiles can promote plant growth but can also increase plant tolerance to stressors (inducing oxidative stress), such as toxic metals, agrochemicals, intense light, high temperature, or emerging pollutants, such as nanoparticles, all of which negatively impact plant growth. The bioactivity of volatiles towards soil bacteria, and in particular PGPR, may enhance their plant growth-promoting activity and support their application, as long as their effects on bacteria are known. In the bioremediation of contaminated sites, the application of volatiles may increase the tolerance of plants and protect microbial communities from pre-existing toxicity, such as Cd toxicity, accelerating the revegetation of polluted sites and reducing the recovery time.

## Data Availability

The data is contained within the article and Supplementary Material.

## Supplementary Materials

The following supporting information can be download at https://www.mdpi.com/article/10.3390/antiox11112303/s1, Table S1: Statistical significance of growth values in *Rhizobium* and *Arabidopsis thaliana* exposed to Cd and 2,3-Butanediol, 3-Methyl-1-butanol and 2-Butanone; Table S2: Statistical significance of biochemical parameters in *Rhizobium* and *Arabidopsis thaliana* exposed to Cd and 2,3-Butanediol, 3-Methyl-1-butanol and 2-Butanone.
