# Supplementary material for: Do Volatiles Affect Bacteria and Plants in the Same Way? Growth and Biochemical Response of Non-Stressed and Cd-Stressed Arabidopsis thaliana and Rhizobium E20-8
Source: Antioxidants (Basel). 2022 Nov 21;11(11):2303. doi: 10.3390/antiox11112303 (PMC9687498; doi:10.3390/antiox11112303)
Supplement: Supplementary file 1 [file antioxidants-11-02303-s001.zip › antioxidants-1959628-supplementary.pdf]

Supplementary Materials

Table S1. Statistical significance of *Rhizobium* and *Arabidopsis thaliana* growth exposed or not to Cd and to different concentrations of 2,3-Butanediol, 3-Methyl-1-butanol and 2-Butanone.

organism/compound/Cd	0nM	1nM	100nM	10uM	1mM	100mM
A. thaliana			mg/ plant			
Rhizobium			mg/ colony			
At-2,3B	3.51±0.62^{a}	3.94±0.08^{a}	3.90±0.13^{a}	3.72±1.17^{a}	2.88±0.99^{a}	2.46±0.34^{a}
At-2,3B-Cd	2.54±1.11^{A}	2.37±0.33^{A*}	2.85±0.95^{A}	2.75±0.52^{A}	2.80±0.33^{A}	3.01±0.44^{A}
Rz-2,3B	89.67±7.91^{a,c}	53.07±9.58^{b,c}	59.73±10.20^{a}	52.61±3.97^{a}	67.48±5.83^{a}	66.19±4.14^{a}
Rzt-2,3B-Cd	58.03±5.92^{A*}	35.80±9.38^{A,C}	29.07±1.40^{A*}	22.90±4.56^{B,C*}	36.55±5.72^{A,C*}	38.30±2.84^{D*}
At-3M	3.51±0.62^{a}	3.87±0.16^{a}	4.06±1.03^{a}	4.99±1.23^{a}	3.71±0.64^{a}	3.47±0.36^{a}
At-3M-Cd	2.54±1.11^{AC}	3.89±1.13^{AC}	3.67±0.32^{A}	3.72±0.73^{B}	2.39±0.34^{C*}	2.54±0.71^{A,C}
Rz-3M	36.20±2.53^{a,c}	38.23±5.20^{a,c}	42.56±4.25^{a}	51.16±4.60^{a,c*}	39.50±1.09^{b,c}	39.73±0.69^{b,c}
Rz-3M-Cd	23.07±0.54^{A*}	30.33±0.95^{B*}	26.77±2.32^{C*}	28.37±2.80^{B,C*}	18.67±0.69^{D*}	15.22±3.18^{D*}
At-2B	3.51±0.62^{a}	4.34±1.35^{a}	2.99±0.51^{a}	3.37±0.58^{a}	3.08±1.02^{a}	2.93±0.21^{a}
At-2B-Cd	2.54±1.11^{A}	3.10±1.08^{A}	3.50±0.54^{A}	3.01±1.30^{A}	2.97±0.45^{A}	2.96±0.27^{A}
Rz-2B	78.57±15.18^{a,c}	91.17±16.30^{b,c}	63.72±7.06^{a}	62.38±5.13^{a}	59.26±9.43^{a}	54.28±17.01^{a}
Rzt-2B-Cd	30.07±3.02^{A*}	41.17±8.12^{A,C*}	35.90±7.66^{A,C*}	36.67±1.74^{B,C*}	34.78±2.50^{A,C*}	19.84±4.12^{D*}

Values are means of at least 3 replicates ± standard error; different lowercase letters indicate significant differences among compounds concentrations in no Cd (0 μM) condition; uppercase letters indicate significant differences among compounds concentrations in Cd (100 μM) condition, and asterisks indicate significant differences between absence (0 μM) and presence (100 μM) of Cd conditions for the same concentration of the same compound. Significantly different values were considered for $p < 0.05$.

Table S2. Statistical significance of biochemical parameters determined in *Rhizobium* and *Arabidopsis thaliana* exposed or not to Cd and to different concentrations of 2,3-Butanediol, 3-Methyl-1-butanol and 2-Butanone.

2,3-Butanediol

organism/compound/Cd	concentration	LPO	PROT	SOD	GPx	GSTs
A. thaliana		umol/mg FW	ug/ mg FW	U/mg FW	mU/mg FW	mU/mg FW
Rhizobium		nmol/g	ug/ mg	U/ mg	uU/mg	uU/mg
**At-2,3B**	0nM	0.11±0.01^{a,c}	48.04±2.10^{a}	0.18±0.00^{a}	7.70±1.52^{a,d}	3.56±0.77^{a,b,c}
	1nM	0.10±0.03^{a,c}	33.81±8.45^{b}	0.14±0.00^{b}	4.51±0.78^{b}	3.86±1.00^{a,b}
	100nM	0.10±0.01^{a,c}	29.00±5.63^{b}	0.15±0.01^{b}	8.78±1.04^{c}	4.04±0.47^{a,b}
	10uM	0.10±0.05^{a}	34.86±2.56^{b}	0.11±0.04^{b,c}	8.58±2.35^{a,c}	6.78±2.07^{c,b,d}
	1mM	0.10±0.02^{c}	65.39±5.53^{c}	0.11±0.02^{c}	11.60±4.05^{a,c}	7.74±2.37^{b,c,d}
	100mM	0.15±0.00^{b}	46.77±3.48^{a}	0.14±00.03^{a,b,c}	24.52±3.82^{d}	7.28±1.46^{c,d}
**At-2,3B-Cd**	0nM	0.15±0.04^{A}	66.14±8.79^{A*}	0.22±0.03^{A}	27.86±1.52^{A*}	38.91±5.00^{A*}
	1nM	0.12±0.04^{A}	55.86±14.47^{A}	0.20±0.01A*	23.42±3.31^{B*}	13.22±1.15^{B*}
	100nM	0.11±0.01^{A}	122.72±9.93^{B*}	0.18±0.04^{A}	11.02±0.89^{B,C,D*}	11.79±1.22^{B*}
	10uM	0.12±0.08^{A}	112.45±19.06^{B*}	0.23±0.07^{A*}	12.64±0.54^{C,D*}	9.18±1.72^{B}
	1mM	0.14±0.03^{A}	90.82±3.32^{B*}	0.24±0.04^{A*}	13.18±7.52^{D}	8.15±1.42^{C}
	100mM	0.17±0.06^{A}	48.28±9.09^{B}	0.06±0.01^{A*}	12.65±0.86^{C,D*}	7.57±1.47^{C}
**Rz-2,3B**	0nM	1.99±0.10^{a}	211.34±83.23^{a}	0.29±0.04^{a,c}	9.04±1.03^{a}	5.20±0.88^{a}
	1nM	4.41±0.66^{b}	188.84±7.48^{a}	0.38±0.03^{b,c}	7.61±0.52^{a,b}	6.43±1.00^{a,b}
	100nM	2.25±0.22^{a}	219.92±32.13^{a}	0.29±0.04^{a,c}	7.64±1.48^{a,b}	5.07±1.08^{a}
	10uM	1.71±0.08^{c}	586.90±82.16^{b}	0.28±0.01^{a}	6.50±1.31^{b}	5.09±1.05^{a}
	1mM	2.28±0.39^{a,c}	203.85±19.56^{a}	0.33±0.04^{c}	6.32±1.31^{b}	6.76±1.09^{a}
	100mM	1.20±0.07^{d}	197.15±60.41^{a}	0.32±0.03^{a,c}	5.80±1.72^{b}	7.31±0.93^{b}
**Rz-2,3B-Cd**	0nM	2.77±0.26^{A*}	359.86±99.59^{A,C}	0.69±0.06^{A*}	13.29±0.18^{A*}	5.80±0.14^{A}
	1nM	3.56±0.32^{B}	356.91±71.04^{A,C}	0.87±0.08^{B*}	12.86±0.53^{B*}	9.48±0.66^{B*}
	100nM	3.25±0.28^{B*}	327.21±69.60^{B}	0.81±0.14^{A,B*}	8.54±2.38^{C}	8.60±2.65^{A}

	10uM	2.23±0.38^{D}	270.69±28.87^{A}	0.64±0.17^{A,B*}	9.68±1.89^{D}	9.25±0.84^{B*}
	1mM	1.23±0.15^{C*}	457.86±97.74^{C}	0.71±0.15^{A,B*}	7.84±1.28^{C,D}	12.97±0.96^{C*}
	100mM	1.97±0.22^{D*}	828.34±53.62^{A}	0.65±0.09^{A*}	19.60±4.07$^{C.D*}$	25.20±3.86^{D*}

2-Butanone

organism/compound/Cd	concentration	LPO	PROT	SOD	GPx	GSTs
A. thaliana		umol/mg FW	ug/ mg FW	U/mg FW	mU/mg FW	mU/mg FW
Rhizobium		nmol/g	ug/ mg	U/ mg	uU/mg	uU/mg
**At-2B**	0nM	0.11±0.01^{a}	48.04±2.10^{a}	0.18±0.00^{a}	7.70±1.52^{a}	3.56±0.77^{a}
	1nM	0.11±0.05^{a}	57.00±2.46^{a}	0.08±0.03^{b}	16.94±5.33^{b}	6.69±2.29^{a}
	100nM	0.13±0.02^{a}	60.32±5.19^{a}	0.14±0.00^{c}	16.88±3.87^{b}	4.53±0.26^{a}
	10uM	0.13±0.03^{a}	85.18±11.60^{a}	0.12±0.02^{b,c}	13.25±0.31^{b}	3.61±1.25^{a}
	1mM	0.15±0.05^{a}	79.28±4.11^{a}	0.11±0.02^{b,c}	22.31±6.17^{b}	4.51±0.71^{a}
	100mM	0.15±0.04^{a}	49.01±4.81^{a}	0.22±0.06^{a}	16.39±3.59^{b}	7.01±0.16^{b}
**At-2B-Cd**	0nM	0.16±0.04^{A}	66.14±8.79^{A*}	0.22±0.03^{A}	27.86±1.52^{A*}	38.91±5.00^{A*}
	1nM	0.17±0.04^{A}	66.50±16.74^{A}	0.20±0.06^{A,B*}	13.97±2.27^{A}	14.00±2.60^{B*}
	100nM	0.14±0.01^{A}	36.83±5.22^{A*}	0.12±0.04^{B}	14.10±5.19^{A}	16.04±9.84^{B,C}
	10uM	0.22±0.09^{A}	36.68±4.33^{A*}	0.20±0.09^{B}	14.95±4.60^{A}	9.84±1.67^{B*}
	1mM	0.22±0.04^{A}	40.25±10.95^{A}	0.21±0.08^{A,B*}	12.23±1.92^{A*}	6.90±0.92^{C*}
	100mM	0.22±0.08^{A}	120.50±46.81^{A*}	0.29±0.09^{A,B}	9.00±1.41^{B*}	27.02±11.95^{A,B*}
**Rz-2B**	0nM	2.04±0.22^{a}	211.34±42.79^{a}	0.29±0.06^{a}	9.04±1.03^{a}	5.20±0.91^{a,b}
	1nM	1.99±0.24^{a}	181.94±25.55^{a}	0.21±0.03^{a}	7.61±0.52^{a,b}	4.84±0.51^{a}
	100nM	2.03±0.39^{a}	260.57±36.72^{b}	0.36±0.05^{a}	7.64±1.48$^{a.b}$	5.38±0.63^{a,b}
	10uM	1.92±0.39^{a}	244.25±58.73^{b}	0.50±0.06^{b}	6.50±1.31^{b}	5.67±0.06^{b}
	1mM	1.87±0.14^{a}	366.57±64.62^{b}	0.52±0.06^{b}	6.19±1.32^{b}	7.57±1.53^{b}
	100mM	2.91±0.35^{b}	350.18±117.23^{a}	0.63±0.08^{b}	5.80±1.72^{b}	7.49±1.66^{b}
**Rz-2B-Cd**	0nM	3.02±0.40^{A*}	359.86±36.54^{A*}	0.69±0.06^{A*}	13.29±0.18^{A*}	5.8±0.49^{A}
	1nM	2.10±0.26^{B}	280.36±50.91^{A,B*}	0.56±0.11^{A*}	12.86±0.53A,^{B*}	3.38±0.08^{B*}
	100nM	2.91±0.60^{A,B}	252.20±14.32^{B}	0.63±0.12^{A*}	9.32±1.43^{B}	5.08±0.77^{A,B}
	10uM	2.54±0.56^{A,B}	250.99±51.07^{B,C}	0.65±0.02^{A*}	9.68±1.89^{B}	5.00±0.82^{A,B}
	1mM	3.58±0.95^{A,B*}	198.51±24.03^{C}	0.53±0.13^{A}	7.84±1.28^{B}	6.95±0.55^{C}
	100mM	11.56±1.09^{C*}	298.00±48.96^{A,B}	1.33±0.07^{B*}	22.93±3.47^{C*}	6.12±0.83^{A,C}

2-Butanone

organism/compound/Cd concentration		LPO	PROT	SOD	GPx	GSTs
A. thaliana		umol/mg FW	ug/ mg FW	U/mg FW	mU/mg FW	mU/mg FW
Rhizobium		nmol/g	ug/ mg	U/ mg	uU/mg	uU/mg
**At-2B**	0nM	0.11±0.01^{a}	48.04±2.10^{a}	0.18±0.00^{a}	7.70±1.52^{a}	3.56±0.77^{a}
	1nM	0.11±0.05^{a}	57.00±2.46^{b}	0.08±0.03^{b}	16.94±5.33^{b}	6.69±2.29^{a}
	100nM	0.13±0.02^{a}	60.32±5.19^{b}	0.14±0.00^{c}	16.88±3.87^{b}	4.53±0.26^{a}
	10uM	0.13±0.03^{a}	85.18±11.60^{c}	0.12±0.02^{b,c}	13.25±0.31^{b}	3.61±1.25^{a}
	1mM	0.15±0.05^{a}	79.28±4.11^{b}	0.11±0.02^{b,c}	22.31±6.17^{b}	4.51±0.71^{a}
	100mM	0.15±0.04^{a}	49.01±4.81^{a}	0.22±0.06^{a}	16.39±3.59^{b}	7.01±0.16^{b}
**At-2B-Cd**	0nM	0.16±0.04^{A}	66.14±8.79^{A*}	0.22±0.03^{A}	27.86±1.52^{A*}	38.91±5.00^{A*}
	1nM	0.17±0.04^{A}	66.50±16.74^{A}	0.20±0.06^{A,B*}	13.97±2.27^{A}	14.00±2.60^{B*}
	100nM	0.14±0.01^{A}	36.83±5.22^{B*}	0.12±0.04^{B}	14.10±5.19^{B}	16.04±9.84^{B,C}
	10uM	0.22±0.09^{A}	36.68±4.33^{A*}	0.20±0.09^{B}	14.95±4.60^{B}	9.84±1.67^{B*}
	1mM	0.22±0.04^{A}	40.25±10.95^{B}	0.21±0.08^{A,B*}	12.23±1.92^{B*}	6.90±0.92^{C*}
	100mM	0.22±0.08^{A}	120.50±46.81^{B*}	0.29±0.09^{A,B}	9.00±1.41^{C*}	27.02±11.95^{A,B*}
**Rz-2B**	0nM	2.04±0.22^{a}	211.34±42.79^{a}	0.29±0.06^{a}	9.04±1.03^{a}	5.20±0.91^{a,b}
	1nM	1.99±0.24^{a}	181.94±25.55^{a}	0.21±0.03^{a}	7.61±0.52^{a,b}	4.84±0.51^{a}
	100nM	2.03±0.39^{a}	260.57±36.72^{b}	0.36±0.05^{a}	7.64±1.48^{a,b}	5.38±0.63^{a,b}

	10uM	1.92±0.39[a]	244.25±58.73[b]	0.50±0.06[b]	6.50±1.31[b]	5.67±0.06[b]
	1mM	1.87±0.14[a]	366.57±64.62[b]	0.52±0.06[b]	6.19±1.32[b]	7.57±1.53[b]
	100mM	2.91±0.35[b]	350.18±117.23[a]	0.63±0.08[b]	5.80±1.72[b]	7.49±1.66[b]
**Rz-2B-Cd**	0nM	3.02±0.40[A*]	359.86±36.54[A*]	0.69±0.06[A*]	13.29±0.18[A*]	5.8±0.49[A]
	1nM	2.10±0.26[B]	280.36±50.91[A,B*]	0.56±0.11[A*]	12.86±0.53[A,B*]	3.38±0.08[B*]
	100nM	2.91±0.60[A,B]	252.20±14.32[B]	0.63±0.12[A*]	9.32±1.43[B]	5.08±0.77[A,B]
	10uM	2.54±0.56[A,B]	250.99±51.07[B,C]	0.65±0.02[A*]	9.68±1.89[B]	5.00±0.82[A,B]
	1mM	3.58±0.95[A,B*]	198.51±24.03[C]	0.53±0.13[A]	7.84±1.28[B]	6.95±0.55[C]
	100mM	11.56±1.09[C*]	298.00±48.96[A,B]	1.33±0.07[B*]	22.93±3.47[C*]	6.12±0.83[A,C]

Values are means of at least 3 replicates ± standard error; different lowercase letters indicate significant differences among compounds concentrations in no Cd (0 μM) condition; uppercase letters indicate significant differences among compounds concentrations in Cd condition, and asterisks indicate significant differences between conditions (0 and 100 μM Cd) for the same concentration of the same compound. Considered significantly different values of $p < 0.05$.
